# Three-dimensional kinematic change of hindfoot during full weightbearing in standing: an analysis using upright computed tomography and 3D-3D surface registration

**DOI:** 10.1186/s13018-019-1443-z

**Published:** 2019-11-11

**Authors:** Kazuya Kaneda, Kengo Harato, Satoshi Oki, Tomohiko Ota, Yoshitake Yamada, Minoru Yamada, Morio Matsumoto, Masaya Nakamura, Takeo Nagura, Masahiro Jinzaki

**Affiliations:** 1 Department of Orthopedic Surgery, Keio University School of Medicine, Tokyo, Japan; 2 Department of Diagnostic Radiology, Keio University School of Medicine, Tokyo, Japan; 3 Department of Clinical Biomechanics, Keio University School of Medicine, 35 Shinanomachi, Shinjuku, Tokyo 160-8582 Japan

**Keywords:** Upright computed tomography, Hindfoot, Weightbearing, Surface registration

## Abstract

**Background:**

Weightbearing of the hindfoot affects positional changes of the ankle joint and subtalar joint (ankle-joint complex [AJC]). However, it is difficult to assess the kinematic changes in the hindfoot in a natural full weightbearing condition using conventional CT or cone beam computed tomography (CT) due to limitations of acquiring foot images under a physiological weightbearing condition using those imaging modalities. Analysis of AJC kinematics using fluoroscopy and 2D-3D registration technique requires data on the number of steps and amount of time to build and match the bones. This study aimed to analyze the effect of full weightbearing on hindfoot motion when standing using upright CT and 3D-3D surface registration.

**Methods:**

Forty-eight AJCs of 24 asymptomatic volunteers (13 women, 11 men) were examined under no weightbearing, 50% weightbearing, and single leg full weightbearing conditions while standing. The CT images were acquired from the distal femur to the whole foot using a 320-row upright CT scanner. The condition of each weightbearing stance was measured using a pressure mat. Bone-to-bone rotations of the talus relative to the tibia and calcaneus relative to the talus were evaluated using the surface registration technique. Image quality of the CT and intra- and interobserver reliabilities of the rotation angle were also evaluated.

**Results:**

All CT images were excellent or good quality and the intra- and interobserver correlation coefficients for the angle were 0.996 and 0.995, respectively. The motion of the ankle joint and subtalar joint under 50% and 100% weightbearing were as follows (in degrees); the talus plantarflexed (5.1 ± 4.5 and 6.8 ± 4.8), inverted (1.3 ± 1.4 and 2.0 ± 1.6), and internally rotated (2.4 ± 4.2 and 4.3 ± 4.6) relative to the tibia, and the calcaneus dorsiflexed (2.8 ± 1.4 and 3.8 ± 1.7), everted (5.3 ± 2.6 and 8.0 ± 3.6), and externally rotated (3.0 ± 2.0 and 4.1 ± 2.4) relative to the talus, respectively.

**Conclusions:**

The effect of weightbearing was clearly identified using an upright CT and the 3D-3D registration technique. Three-dimensional kinematics under static full weightbearing were opposite between the ankle and subtalar joints on their respective axes.

## Background

The joint of the hindfoot, i.e., the ankle-joint complex (AJC), consists of the ankle joint and the subtalar joint. One of the major functions of the AJC is adjusting the lower limb alignment while weightbearing. It is difficult to assess the kinematic change of the AJC under the weightbearing condition using 2D radiography because of the complex bony shapes of the joint. Thus, the 2D-3D registration technique was developed to measure foot bone and AJC kinematics of patients [1, 2] or asymptomatic volunteers [3–6]. Although the accuracy of the 2D-3D registration technique is below 1.0 mm and 1.0°, the number of steps is required to achieve accuracy. First, computed tomography (CT) scans are crucial to build the bone model. Second, 2D fluoroscopic images require calibration to adjust enlarged images projected from radiographs. Finally, a matching algorithm involves a number of mathematical calculations and optimization. Therefore, a method to analyze the kinematics of AJC without such time and cost is needed.

Many studies have analyzed the effect of weightbearing on the hindfoot using magnetic resonance imaging [1] or conventional CT with loading devices [7–15] or upright cone beam CT [16–23]. However, the effect of natural full weightbearing in a standing position has not been evaluated due to limitations of acquiring foot images under a physiological weightbearing condition using those imaging modalities. Major limitations of these modalities include low resolution and longer scanning time (MRI), prone position and non-physiological weightbearing (conventional CT), and motion artifact and partial weightbearing (cone beam CT). Approximately 1 to 2° of bony motion were observed in the AJC under weightbearing [10, 11, 15], while no study has reported the kinematic change in the AJC between non weightbearing and full weightbearing positions. Recently, we developed an upright CT with an area detector with Canon Medical Systems [24], in which a CT scan under full weightbearing in a natural standing condition can be acquired.

The present study aimed to analyze the effect of full weightbearing on hindfoot motion of asymptomatic feet using an upright CT with a 320-row multidetector and 3D-3D surface registration technique. We hypothesized that the upright CT and 3D-3D registration technique clearly reveals kinematic change in the AJC due to natural full weightbearing.

## Materials and methods

### Subjects

A total of 48 AJCs of 24 healthy volunteers (13 women, 11 men) with no history of a foot or ankle injury and no obvious foot deformations were enrolled in the present study. The mean (± standard deviation) age, body weight, and body mass index (BMI) of the participants were 28.3 ± 4.0 (range, 23–39) years, 59.0 ± 9.7 (range, 45.0–78.0) kg, and 21.4 ± 2.0 (range, 17.6–26.4) kg/m^2^, respectively. Each participant provided written informed consent, and the study protocol (ID#20150293) was approved by our ethical committee.

### Image acquisition

**Fig. 1:**
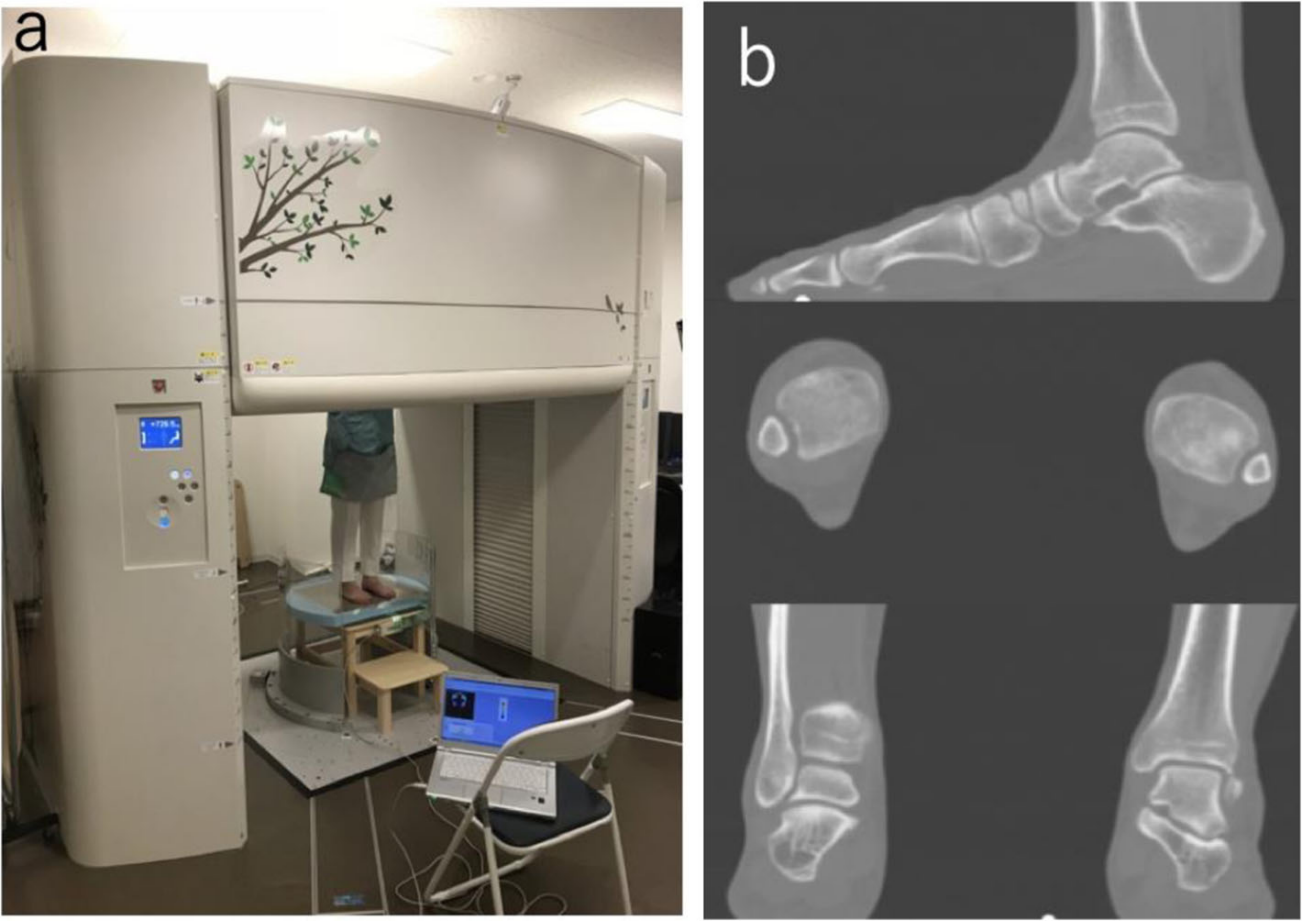
320-row upright computed tomography (CT) scanner. **a** The CT images were acquired from the distal femur to the entire foot using a 320-row upright CT scanner (prototype TSX-401R; Canon Medical Systems, Otawara, Japan). The condition of each weightbearing stance was measured using a pressure mat (BIG-MAT; NITTA Corporation, Osaka, Japan) and pressure calculation system (FootMat; Tekscan, South Boston, MA, USA). **b** The image qualities of the upright CT scanner are good to excellent

The CT images were acquired from the distal femur to the entire foot using the 320-row upright CT scanner (prototype TSX-401R; Canon Medical Systems, Otawara, Japan) (Fig. 1) [24]. The CT examinations were performed using the following parameters: peak tube voltage, 100 kV; tube current, 10 to 350 mA (using a noise index of 15 for a slice thickness of 5 mm); rotation speed, 0.5 s; and slice thickness, 0.5 mm. No weightbearing, standing (50% weightbearing), and single leg full weightbearing (100% weightbearing) were assessed for each participant [25]. The condition of each weightbearing stance was measured using a pressure mat (BIG-MAT; NITTA Corporation, Osaka, Japan) and pressure calculation system (FootMat; Tekscan, South Boston, MA, USA). In an upright CT scanner, all participants stood in a relaxed position with their bare feet shoulder width apart. Similar to the previous report, 2-kg weightbearing with the ankle in a neutral position was defined as the no weightbearing condition in the present study [26], while the opposite side was defined as full weightbearing. The CT data were accumulated using the Digital Imaging and Communication in Medicine (DICOM) data format. Two orthopedic surgeons with 8 and 19 years of experience diagnosing musculoskeletal CT images independently evaluated the image quality of all the images using the nine-level Likert scale, as described in the previous study [27]: a score of 5 indicates “excellent” image quality without any artifacts; score of 4 indicates “good” image quality with minor artifacts; score of 3 indicates “fair” image quality with moderate artifacts; score of 2 indicates “poor” image quality with non-diagnostic quality and the visualization task can be identified; score of 1 indicates “very poor” image quality with non-diagnostic quality and the visualization task cannot be identified. A half score was added between each score and a total of nine grades were evaluated.

### 3D-3D surface registration

Three-dimensional surface data of the tibia, talus, and calcaneus were extracted from the CT DICOM data using 3D visualization software (AVIZO 6.4; Thermo Fisher Scientific, Tokyo, Japan). We matched the 3D surface of the talus in each weightbearing condition for each participant using the iterative closest point algorithm using Visualization Toolkit 8.1.0 (Kitware Inc., Clifton Park, NY, USA) for the 3D surface registration technique in which point data are superimposed onto another 3D surface by iterative steps to reach the closest points.

### Coordinate system

**Fig. 2:**
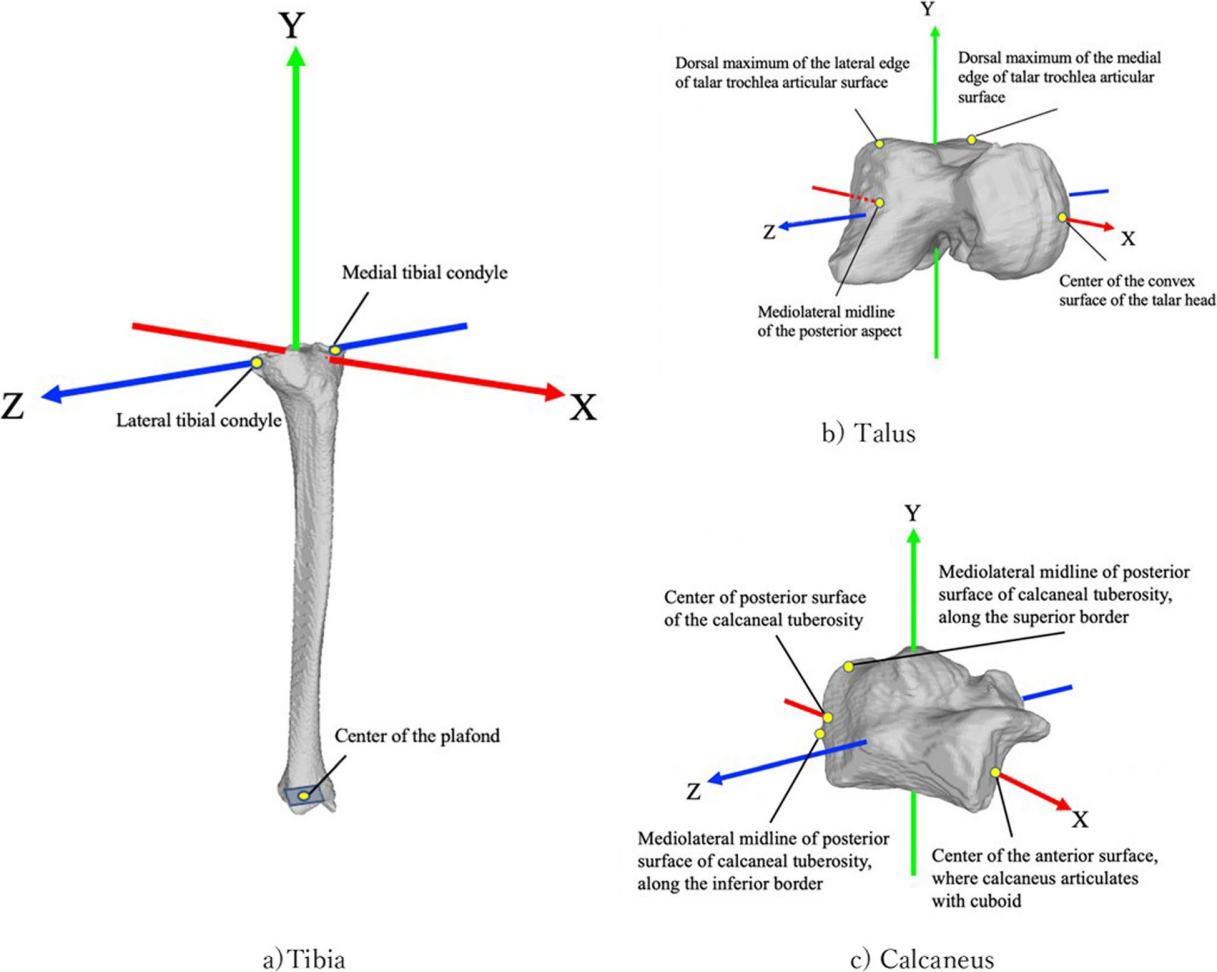
Coordinating system of each bone. **a** The cordinate system of the tibia. **b** The cordinate system of the talus. **c** The cordinate system of the calcaneus. The coordinate system of the tibia was defined as reported by Sato et al. and the International Society of Biomechanics. The coordinate systems of the talus and calcaneus were defined using the method described by Gutekunst et al.

We modified and used the coordinate system of the tibia using the method defined by Sato et al. [28] and the International Society of Biomechanics [29]. The coordinate systems of the talus and the calcaneus were defined using the method described by Gutekunst et al. [30] (Fig. 2).

The tibia coordinate system consisted of the following: the line connecting the center of the most medial point on the border of the medial tibial condyle (MC) and the most lateral point on the border of the lateral tibial condyle (LC) and the center of the tibia plafond pointing upward was defined as the Y axis. The line connecting the MC and LC was defined as the T (temporary) axis. The Z axis was the line perpendicular to the Y and T axes pointing laterally. The X axis was defined as the line perpendicular to the Y and Z axis pointing anteriorly.

The talus coordinate system consisted of the following: the line connecting the mediolateral midline of the posterior aspect of the talus and the center of the convex surface of the talar head centered both mediolaterally and vertically and pointing anteriorly was defined as the X axis. The line connecting the dorsal maximum of the medial edge of talar trochlea articular surface and the dorsal maximum of the lateral surface was defined as the T axis. The Y axis was the line perpendicular to the T and X axes pointing upward. The Z axis was defined as the line perpendicular to the X and Y axes pointing laterally.

The calcaneus coordinate system consisted of the following: the line connecting the midpoint of the posterior surface of the calcaneal tuberosity, centered both mediolaterally and vertically, and the center of the anterior surface of the calcaneus where it articulates with the cuboid pointing anteriorly was defined as the X axis. The line connecting the mediolateral midline of the posterior surface of the calcaneal tuberosity along the inferior border and the mediolateral midline of the posterior surface of the calcaneal tuberosity along the superior border was defined as the T axis. The Z axis was the line perpendicular to the T and X axes pointing upward. The Y axis was defined as the line perpendicular to the X and Y axes pointing laterally.

### Analysis of joint motion

The ankle (talocrural) joint: the articulation formed between the talus and the tibia/fibula. The subtalar joint: the articulation formed between the talus and the calcaneus.

Rotation around the X axis was defined as inversion/eversion, rotation around the Y axis was defined as internal/external rotation, and rotation around the Z axis was defined as dorsiflexion/plantarflexion. Bone-to-bone rotations of the talus relative to the tibia and the calcaneus relative to the talus around each axis are described by the Euler/Cardan angles representing three sequential rotations about the anatomical axis of the proximal bone. The rotation sequence “Z-X-Y” was used.

### Statistical analysis

The intra- and interobserver reliabilities were independently assessed by two orthopedic specialists and by reassessment of the data with an interval of longer than 2 months, respectively. The correlation coefficients were calculated to assess the intra- and interobserver reliabilities using SPSS ver. 24.0 (IBM, Armonk, NY, USA).

## Results

Image qualities of the 144 AJC scans of 24 subjects were good (diagnostic quality with minor artifacts) or excellent (diagnostic quality without any artifacts) [29] (Table 1). The intra- and interobserver correlation coefficients for the present study were 0.996 (95% confidence interval, 0.994–0.998) and 0.995 (95% confidence interval, 0.992–0.997). These data indicated that the present measurement was highly reliable.

**Table 1:** Count of observers rating for the image visibility and artifacts

Score	Number of CT image	
	Observer 1	Observer 2
5: Excellent	108	130
4.5	34	10
4: Good	2	4
3.5	0	0
3: Fair	0	0
2.5	0	0
2: Poor	0	0
1.5	0	0
1: Very poor	0	0

Assessment of all images was performed independently by two observers. A score of 5 (“excellent”) indicates diagnostic quality without any artifacts; score of 4 (“Good”) indicates diagnostic quality with minor artifacts; score of 3 (“Fair”) indicates diagnostic quality with moderate artifacts; score of 2 and 1 (“Poor” and “Very poor”) indicates non-diagnostic quality. Good to excellent motion artifact were found in AJC images with upright CT in present study

**Fig. 3:**
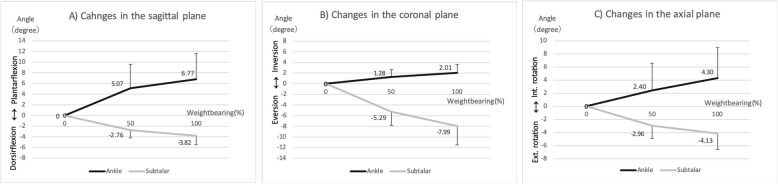
Rotation changes in each plane. Rotational movement of the ankle and subtalar joint in the sagittal, coronal, and axial planes were indicated in **a**–**c**, respectively. In the ankle joint, the talus plantarflexed, inverted, and internally rotated relative to the tibia as the weightbearing increased. Conversely, at the subtalar joint, the calcaneus dorsiflexed, everted, and externally rotated relative to the talus as the weightbearing increased

**Fig. 4:**
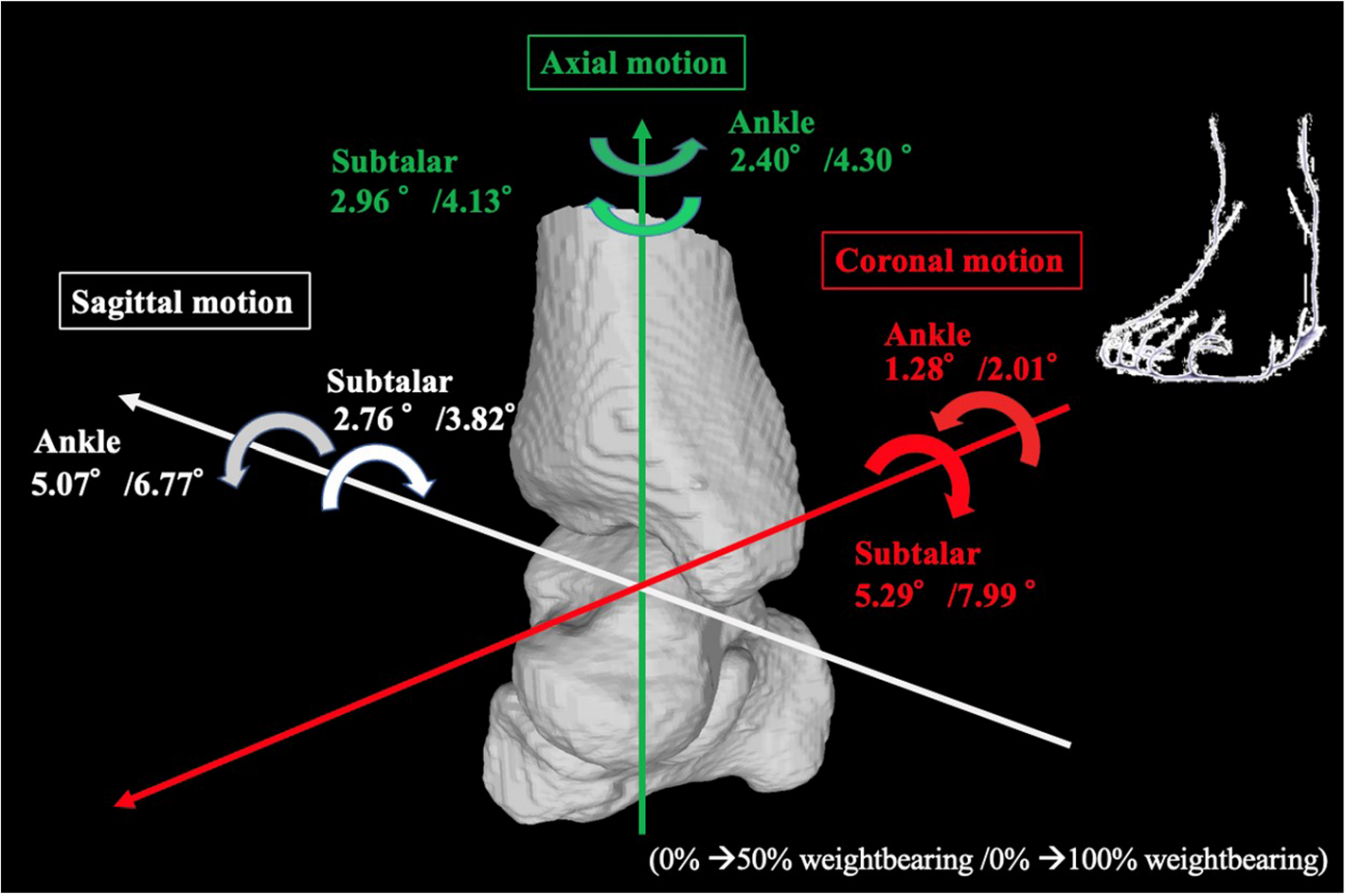
Hindfoot kinematics of the right foot during 50% and 100% weightbearing. The numbers indicate motion of the joints during 0% → 50% weightbearing/0% → 100%weightbearing. Paradoxical movement between the ankle and subtalar joints occurs as the talus plantarflexed, inverted, and internally rotated relative to the tibia and the calcaneus dorsiflexed, everted, and externally rotated relative to the talus as the weightbearing increased

Figure 3 shows the amount of change in each direction under each condition, and Fig. 4 summarizes the movement directions, with full weightbearing in one figure.

In the ankle joint, the talus plantarflexed (50%/100% weightbearing, 5.07 ± 4.52/6.77 ± 4.84 degrees), inverted (50%/100% weightbearing, 1.28 ± 1.37/2.01 ± 1.58 degrees), and internally rotated (50%/100% weightbearing, 2.40 ± 4.18/4.30 ± 4.64°) relative to the tibia as the weight load increased. Conversely, at the subtalar joint, the calcaneus dorsiflexed (50%/100% weightbearing, 2.76 ± 1.42/3.82 ± 1.68°), everted (50%/100% weightbearing, 5.29 ± 2.56/7.99 ± 3.55°), and externally rotated (50%/100% weightbearing, 2.96 ± 1.95/4.13 ± 2.43°) relative to the talus as the weight load increased (Figs. 3 and 4). Three-dimensional kinematics were opposite between the ankle joint and the subtalar joint on their respective axes, and each angle increased as the weight load increased. Regarding the absolute value, sagittal and axial plane movements were larger in the ankle joint, while the coronal plane movement was larger in the subtalar joint.

## Discussion

Our approach using the upright CT and 3D-3D registration technique clearly described the effect of full weightbearing in AJC kinematics, and the results support our hypothesis.

The bony motions in the AJC under weight load in the past studies [10, 11, 15] were lower than those in the present study, likely because of insufficient and unphysiological weightbearing (Table 2).

**Table 2:**
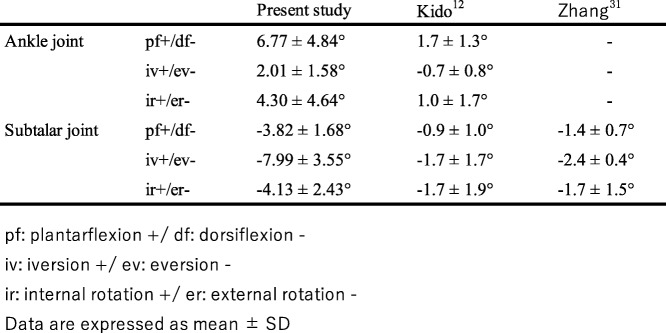
Comparison of the hindfoot kinematics with past studies

Due to the simulated weightbearing conditions, there were limitations in the hindfoot kinematics in previous studies. The direction of rotation was different from the present study and the values in their studies were also lower than those in the present study

Our method to analyze AJC kinematics has several advantages over the methods using fluoroscopy or other imaging modalities (Table 3). First, 3D-3D registration on CT images requires fewer steps to match the bone and evaluate AJC kinematics and it is easier to match 3D to 3D than 2D to 3D models. Analysis of foot bone and AJC kinematics using fluoroscopy and the 2D-3D registration technique has been reported [1–6]; however, its major limitation is the complex nature of the steps required to build and match the bones. The 2D images taken by fluoroscopic imaging are shadow pictures, and a 3D bone model based on CT images is required to accurately match the bones on the 2D images. Image calibration is also required to adjust enlarged images when using the X-ray system. Several matching algorithms have been developed, but the significant time and cost required to analyze the kinematics of the bones limit its use. The accuracy of the 3D-3D registration was below 0.2° in rotation [31]. Second, only minor motion artifacts were found in AJC images with upright CT in the present study (Table 1). Changes in hindfoot alignment have been assessed using upright cone beam CT [16–23], but it takes as long as 20 to 48 s to acquire images, and it is necessary for participants to support the body to reduce artifacts. In fact, moderate to severe motion artifacts were observed in the cone beam CT images of the knee and ankle [27]. In addition, participants must put their foot in a small tube of the cone beam CT, and thus the participants must set their contralateral foot somewhere aside from the tube or stabilize their body using supportive tools such as a pole. This position is not a natural standing position, and only partial weight is loaded on the foot. Third, physiological weightbearing while standing can be acquired in the upright CT, while simulated weight with loading devices was applied in the studies using conventional CT [7–15]. In those studies, the hip, shoulder, or knee must be fixed to reproduce the hypothetical loading conditions, and the lower limb muscles used to maintain the standing position was not active in the prone position. Those limit the representation of physiological loading and tarsal bone alignment while standing.

**Table 3:** Comparison of the methods to analyze the hind foot kinematics

Methods	Image quality	Image acquisition time	Matching algorithm	Weightbearing
Fluoroscopy and 2D-3D registration	2D	Several seconds	2D-3D/image calibration and optimization	Full
Cone beam CT	3D/motion artifact	20–48 s	3D-MPR/evaluate only in 2D plane	Full/partial
Conventional CT	3D	10–20 s	3D-3D/volume marge technique	Simulated
MRI	3D/motion artifact	120–180 s	3D-3D/marching cubes method	Full/partial
Upright CT and 3D-3D registration	3D	10–20 s	3D-3D/iterative closest point	Full

List of the methods to analyze the hind foot kinematics. There are differences in image dimension/quality, acquisition time, algorithm, and weightbearing condition

Several limitations of the present study should be noted. First, there were no patient data, and only asymptomatic subjects were included. However, our method using an upright CT and 3D-3D registration technique can be a powerful tool to investigate kinematic change in the AJC of the patients. The clinical relevance of the hindfoot motion during natural full weightbearing should be studied in the near future. Second, the imaging was divided into three categories, i.e., no weightbearing, 50% weightbearing, and full weightbearing, and static imaging was performed. Although continuous imaging in 4D was possible using an upright CT with 320-row multidetector, the image quality of 4D CT was insufficient to capture the tarsal bones; thus, we separately scanned the three loading conditions. To analyze the continuous dynamics of the hindfoot, we need to increase the observation points under different weightbearing conditions in a future study.

## Conclusion

An upright CT and 3D-3D registration technique clearly described the kinematics of the AJC in a static full weightbearing condition. Our findings demonstrated that 3D motions were opposite between the ankle and subtalar joints on their respective axes.

## Data Availability

The datasets of the present study are available from the corresponding author on reasonable request.

## Abbreviations

AJC: Ankle-joint complex
CT: Computed tomography
DICOM: Digital Imaging and Communication in Medicine
LC: Lateral tibial condyle
MC: Medial tibial condyle
